# Measuring for Success: Evaluating Leadership Training Programs for Sustainable Impact

**DOI:** 10.5334/aogh.3221

**Published:** 2021-07-12

**Authors:** Joel Njah, Bhakti Hansoti, Adebusuyi Adeyami, Kerry Bruce, Gabrielle O’Malley, Mary Kay Gugerty, Benjamin H. Chi, Nanyombi Lubimbi, Elizabeth Steen, Sonora Stampfly, Eva Berman, Ann Marie Kimball

**Affiliations:** 1 ICAP, Columbia University, USA; 2 Afya Bora Consortium; 3 Johns Hopkins School of Medicine, STAR Program, USA; 4 King’s College London, London, United Kingdom; 5 Clear Outcomes/WomenLift Health, USA; 6 Department of Global Health, University of Washington, Seattle, USA; 7 Daniel J. Evans School of Public Policy & Governance, University of Washington, International Program in Public Health Leadership, Seattle, USA; 8 University of North Carolina at Chapel Hill, USA; 9 College of Nursing, University of Illinois at Chicago, Chicago, USA; 10 Emerita, University of Washington; 11 Chatham House

## Abstract

**Background:**

In an era of global health security challenges such as the COVID-19 pandemic, there is greater need for strong leadership. Over the past decades, significant investments have been made in global health leadership development programs by governments and philanthropic organizations to address this need. Evaluating the societal impact of these programs remains challenging, despite consensus on the importance of public health leadership.

**Objective:**

This article identifies the gaps and highlights the critical role of monitoring and evaluation approaches in assessing the impact of global health leadership programs. Importantly, we also propose the theory of change (TOC) as a common framework and identify a set of tools and indicators that leadership programs can adapt and use.

**Methods:**

We carried out an informal review of major global health leadership programs, including a literature review on leadership program evaluation approaches. Current practices in assessing the short- to long-term outcomes of leadership training programs were explored and synthesized. We also examined use of program theory frameworks, such as theory of change to guide the evaluation strategy. We find the TOC approach can be enhanced by integrating evaluation-specific frameworks and establishing broad stakeholder buy-in. We highlight measurement challenges, proposed outcome indicators and evaluation methodologies, and outline the future direction for such efforts.

**Findings:**

Most evaluation of current leadership programs is focused on short-term individual-level outcomes, while reports on long-term societal impact were limited. Reciprocal impacts on and benefits for the “host” organizations were not included in evaluation metrics. Most programs had program logic or result chains, but with no well-articulated program theories.

**Conclusion:**

Key stakeholders involved in leadership training programs benefit from the evidence of rigorous program evaluations to inform decisions that address barriers in fostering global health leadership and improving population health outcomes. Insight into reciprocal change in host organizations is important. Evaluation of global health leadership training must go beyond the individual trainee and encompass organizational and community-level impacts. Documentation of long-lasting organizational and societal impacts is essential for donors to appreciate the return on their investment.

**Key Takeaways:**

- Evaluation plays an important role in understanding how leadership development takes place and how it contributes to improving public health outcomes.
- Making the case for investments in leadership development programs requires robust evidence from monitoring and evaluation strategies that link investments beyond the individual-level to longer-term societal impacts.
- The first critical step towards a strategy for success is for leadership programs to clearly build, articulate, share, and use their program theories or theories of change.
- Theories of change help identify the pathways (and potential tensions) through which leadership development programs effect change at the individual, organizational and community levels.
- Evaluation methods that examine outcomes of leadership programs should be multi-method, multi-level, and where possible include counterfactual outcomes.
- Allocation of funds to evaluate on-going and long-lasting societal impact of leadership programs should be a routine practice.

## Introduction

In today’s constantly changing landscape, new infectious, environmental, and behavioral challenges, such as COVID-19, Ebola and Zika, highlight the need for strong leadership. Significant investments have been made in leadership training and several training programs have emerged to address this need. Public health leadership has been identified as an imperative for global health security [1]. In 2010, The Lancet Commissioners identified weak leadership as one of the global systemic failures in improving health-system performance in the 21st century [2]. More recently, the former Minister of Health for Rwanda stated, “thoughtful leadership and effective management practices are necessary to strategically and equitably improve health systems” [3].

Monitoring and Evaluation (M&E) is used to assess the performance of projects, institutions, and programs set up by governments, philanthropy, international organizations, and non-governmental organizations (NGOs). The goal of M&E is to improve current and future management of outputs, outcomes and impact. At the program level, the purpose of M&E is to track implementation and outputs systematically. At the donor/funder level, M&E reports are used to determine success or advocate for refunding and are an important part of accountability to funding agencies and stakeholders. Given the underlying objectives of many global health leadership programs, such as improved health equity and health outcomes, M&E strategies that focus on simple metrics, such as numbers trained, paint an overly simplified view of leadership program progress and success.

The pathways through which the increased leadership capacity of individuals leads to organizational and ultimately societal impact are tenuous. Most programs measure the “success” of leadership programs, by measuring process outputs anchored to the program objectives and goals as opposed to the true long-term impact of the program on global health. These M&E efforts are typically immediate or short-term in nature. Few M&E frameworks capture the impact of the trainee on host institutions or global health outcomes more broadly.

Identifying measures of downstream organizational or societal impact is essential to meaningful assessment and improvements in global health leadership training programs. This requires a thoughtful consideration of the catalytic impact a training program has on individuals, their institutional or policy arena, and ultimately on health systems. Training leaders is complex and thus measuring evidence of their impact is challenging. This paper reviews the current practices of assessing the success of global health leadership programs and outlines potential strategies that go beyond assessment to measuring impact.

This paper contributes to the limited body of research on measuring and assessing the impact of leadership training programs by proposing a common framework—theory of change (TOC)—and a set of tools and indicators that leadership program designers can adapt to fit across programs. We review a selected sample of leadership training programs that are either practice or policy focused and propose a set of anticipated outcomes, potential indicators, and methods of data collection. Lastly, we discuss the impediments and solutions to measuring long-term impact on and reciprocal benefits to organizations hosting these programs.

## Current Evaluation Strategies for Leadership Training Programs

In our review of the literature, we found that evaluation has focused on measuring individual-level outcomes with some recent attention to leadership outcomes at the organizational level [4]. Leadership program directors and funders need to plan for measurement at the outset of the program, with short-, medium-, and long-term impacts, across socio-ecological levels that adequately capture change over time. The preoccupation with short-term impacts at the individual level represents a serious gap in evaluation practice, theory and design. Reevaluating the changes individuals experienced 2–10 years after completion of a program offers an opportunity to assess whether improvements are lasting and provides a chance to understand whether other unintended results have emerged over time [5]. Unfortunately, post-project reevaluation is rare; relatively few development projects are evaluated after funding ends. Even less common are systematic reviews of the culture shifts, learning, and insights gained by organizations executing these programs.

There are over 200 differing ideas and theories on leadership. ***Table 1*** presents a relevant overview of the major approaches to the measurement of impact of public health training programs.

**Table 1:** Measuring Impact in Selected Public Health Training Programs.


TITLE (YEAR)	EVALUATION STRATEGIES

Levels of Evaluation: Beyond Kirkpatrick (1994) [6]	Expands the Kirkpatrick Model by adding a fifth level concerned with societal impact and by slightly redefining some of the levels to apply to human performance interventions in general.

The Bass handbook of leadership: Theory, research, and managerial applications, 4th ed.(2004) [7]	The foundational approach to measuring training programs, Kirkpatrick’s Four-Level Model, first introduced in 1959. The four levels of the model include 1) reaction, 2) learning, 3) behavior, and 4) results.

The Results of an Evaluation Scan of 55 Leadership Development Programs (2004) [8]	Most assessments do not move past the first two levels, reaction and learning, stopping at program satisfaction and knowledge gained.

Management Matters: A Leverage Point for Health System Strengthening in Global Health (2015) [9]	An overview of about 2 dozen studies examining the link between management and health system performance. Results showed that training interventions can have influence, but none showed causal relationship between training and health outcomes. This review is limited in “size, scope and rigor” with a focus on process indicators, levels of satisfaction, and knowledge gained on self-reported set of specific competencies.

Evaluating the Impact of Leadership Development (2017) [10]	Evaluation of leadership interventions is evolving: a growing body of work that sees leadership as a “networked process.” A shift from competency-based developed to vertical development that supports thinking in more complex, systematic, and strategic ways. It emphasizes cultural responsiveness.

Using Social Network Analysis in Evaluation (2013) [11]	Social Network Analysis in evaluation is useful when the leadership initiative is expected to lead to observable changes in a network structure. This tool can help understand the network embedded within a program or initiative, in terms of its density, connectedness, balance, and/or centralization.

Measuring Leadership development: Quantify your program’s impact and ROI on organizational performance (2012) [12]	Return on investment (ROI) approaches to evaluating leadership development connect leadership development strategy and activities to a specific mission. The ROI approach focuses first on measuring individual reactions, learning and behaviors, then seeks to connect those changes directly to specific, measurable objectives, such as measures of improved patient or community health outcomes.


### Survey of Current Programs

The evaluation of leadership initiatives has varied greatly in design and approach. There is no “gold standard” by which to assess success. In an informal survey of the programs led by the study authors, we found that few metrics were used consistently, making it difficult to assess relative impact of leadership training across programs. However, we noted several trends: 1) Most programs had a heavy emphasis on short-term (1–2 year) outputs. These metrics often focused on individual trainees, with emphasis on new knowledge/skills gained and early accomplishments (e.g., projects completed, grants awarded). Some focused on the program outputs as well (e.g., number of individuals that completed training, new program curricula developed and implemented). 2) As programs described their evaluation metrics over a lengthier time scale, however, the number and specificity of measurement indicators typically decreased. In the medium-term horizon (i.e., 2–5 years), programs often focused on how individuals interacted with their work environment, including behavioral changes, performance, networking, and organizational capacity building. When the scope was extended to the long-term (>5 years), greater emphasis was placed on broader institutional changes across multiple institutions and sectors. 3) We identified only two projects that measured organizational level and/or societal level outcomes, but one is still in the early (1–2 year) stage.

## Current Approaches to Leadership Program M&E

**Figure 1:**
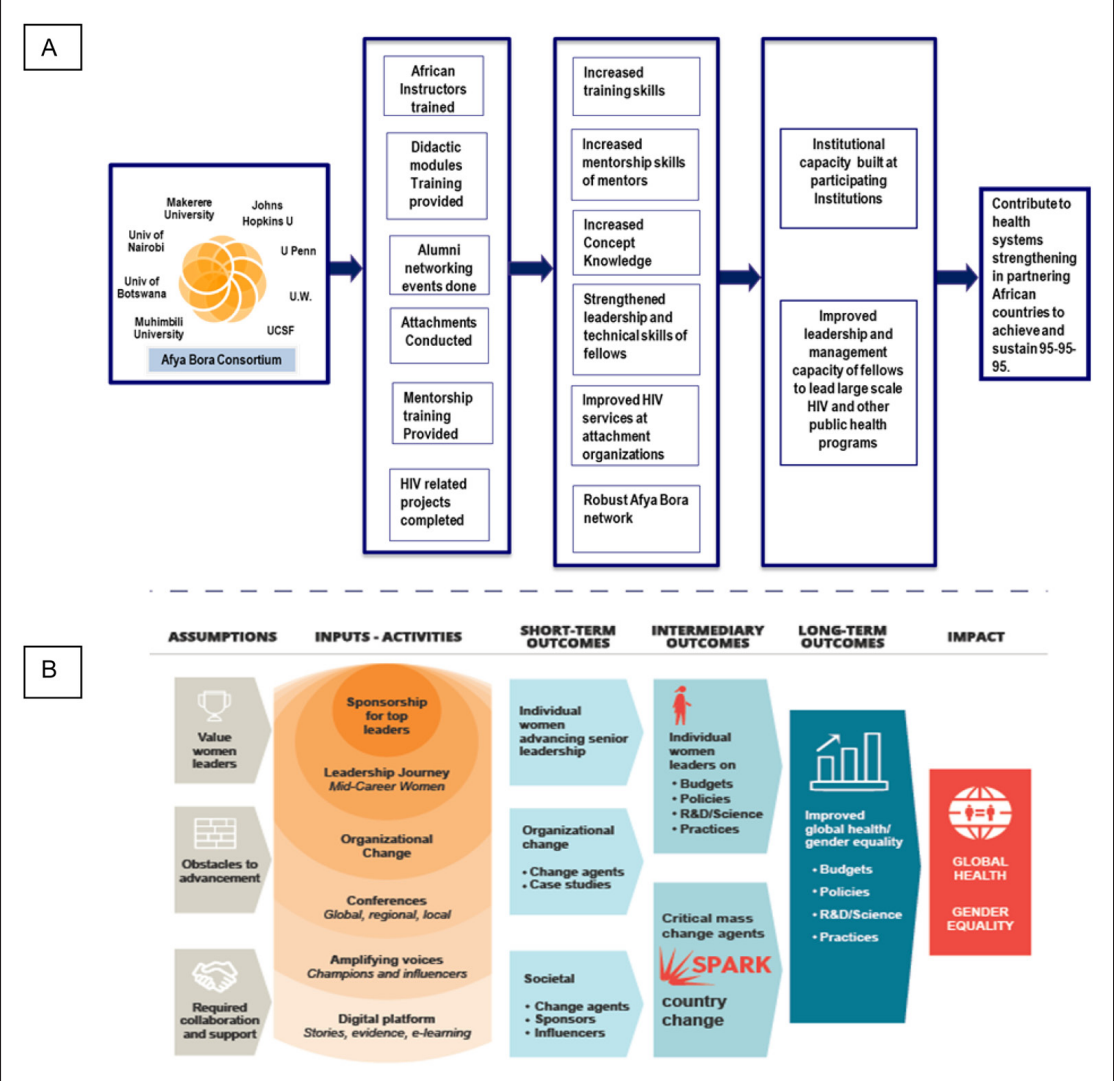
Examples of program logic models: **Panel A**—Afya Bora Consortium program logic model and **Panel B**—The WomenLift Health Initiative program logic model.

There is a large body of literature that demonstrates the critical role of theories and frameworks in guiding and explaining how to evaluate the outcomes of complex public health leadership programs for improved global health [13,14]. One of the first steps is to develop a logic model that demonstrates the underlying **theory of change** for leadership intervention, describing what the leadership training program is trying to achieve and how it hopes to get there. This is a crucial, antecedent step in designing an evaluation strategy and illustrative examples of effective logic models are included in ***Figure 1***.

**Figure 2:**
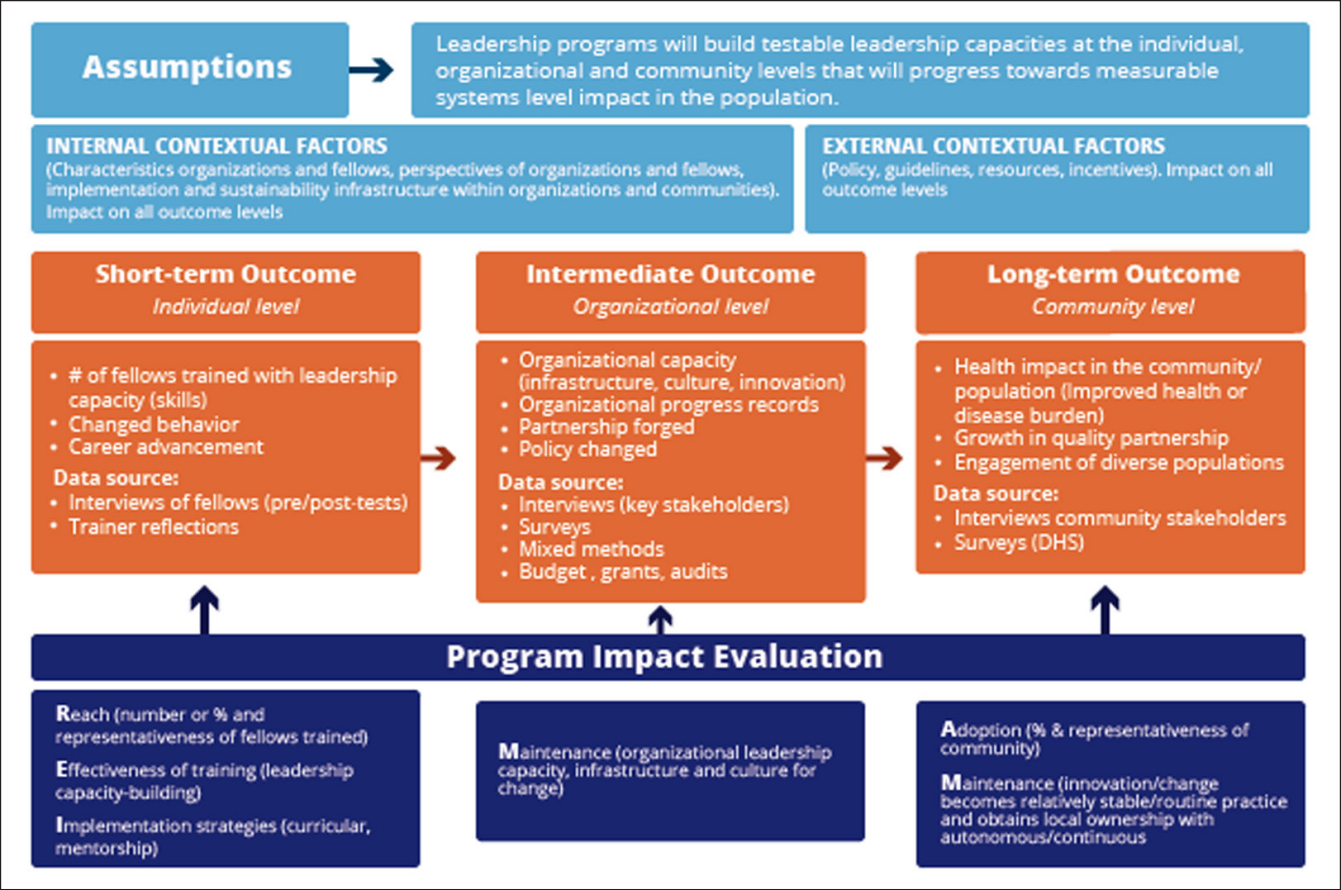
Sample Theory of Change framework for Leadership Program Evaluation.

Once the leadership program’s key components have been identified and its theorized causal pathways articulated, decisions can be made about which components and pathways of the program are of most interest. A TOC can also facilitate broad stakeholder buy-in and agreement, as well as guide the allocation of evaluative resources. The challenge then lies in identifying and then applying measurable indicators to components identified as critical to the pathway of change. The field of implementation science has several established evaluation specific indicators that have well defined measurement metrics. In ***Figure 2*** we present both a TOC framework and provide illustrative examples of measurement indicators (from the RE-AIM evaluation framework) that could be applied.

## Guiding Principles in Public Health Leadership Measurement

To understand the comprehensive effect of public health leadership programs, we need to invest in the measurement of both short-term and long-term outcomes. As an evaluation shifts from short-term to long term-outcomes, so does the socio-ecological level of measurement which will progress from the individual to organizational and community/societal levels. In ***Table 2*** we propose sample indicators and evaluation strategies.

**Table 2:** Short-, medium- and long-term indicators and measurement strategies.


INDICATORS	EVALUATION METHODS

Short-term (1–2 years)	

Largely outputs (number of participants, demographics, sessions, content, trainers, time) Self-reported change in knowledge, skill, point of view, awareness Size, strength of networks	Routine monitoring of outputs Self- assessment and 360 Surveys of participants and relevant actors Key informant interviews with participants and relevant actors Social network analysis (if relevant)

Medium-term (3–5 years)	

Achievement of new leadership positions Continued behavior change in leaders (risk-taking, collaboration) Growth of networks Nascent organizational changes	Everything above and, Review of organizational change caused by participation in leadership programs Comparison of cohorts over time Tracking of career outcomes Use of qualitative methods to understand outcomes

Long-term (5+ years)	

Change in implementation of policy, practice Culture of learning and collaboration Organizational changes Value changes Sustainability Health outcomes	Everything above and, Research into relevant indices and tracking societal level change over time Differences in differences analyses between cohorts or between cohorts and counterfactuals Contribution analysis Sustainability assessments Use of qualitative methods to understand sustainability at the community level


## Measurement Design Considerations

### Challenges of Measurement

Evaluating leadership programs is inherently complex. Leadership development and leadership impact are characterized by dynamic and interlinked processes of change. Leadership development—whether at the individual, organizational or societal level—is subject to multiple feedback loops that change the conditions in which the training operates. The leadership development process continues well beyond typical high-touch points of the intervention (e.g., beyond a one-year fellowship training program). While positive leadership development at the individual level may be evident/observable during a training program, greater individual leadership growth is likely to take place multiple years out. This is the result of the accelerator effect of better learning through successive leadership experiences, the cumulative effect of broader network development with similarly trained leaders, or the catalytic effect of engagement of additional leadership mentors.

Leadership evaluation uses a number of methods to measure the immediate impact of leadership programs on individual leadership abilities, including the use of 360 evaluations, assessment of self-efficacy, pre-post training assessment based on the Kirkpatrick model. Leadership development, however, is also strongly impacted by an individual’s organizational environment, including the incentives of an organization and the type of leadership behaviors it rewards. Long-term leadership evaluation has often struggled to adequately specify and measure how context affects outcomes. Doing so can illuminate how and when improved leadership in individuals translates into improved organizational and system-level outcomes— the contextually situated process of leadership.

Social network analysis can also expand the range of results beyond individual outcomes to reveal the pattern and strength of relationships formed as the result of a leadership training [15]. Network maps can reveal important hubs and bridges that are critical to the flow of ideas and catalysts for change [16]. Networks can be examined at the peer/team, organizational, field, and collective leadership levels, as well as, evaluating for connections among those levels [15].

### Moving from Attribution to Contribution

Evaluation of leadership programs beyond the individual level tends to face several, inter-related challenges. The narrower and more upstream the evaluation focus, the easier and clearer the attribution of the leadership program (e.g., change in knowledge, skills, attitudes). The further beyond the individual level of measurement one goes (the downstream impact), the longer the timeframe to see the intended changes the leadership program aimed for. Moreover, when leadership development programs aim to influence outcomes beyond the organizational level, the theory of change needs to articulate the specific changes intended at multiple levels, which might include community, network, field, movement, population, and systems. These intended changes would need to be explicitly aligned between the executing or “training” program and the organization to which the participant is returning. The further downstream one looks for impact, the wider the field of contributing factors to that impact (Figure 3). This challenge is inherent in evaluating any leadership program, most training programs, and other complex interventions. Given these challenges and complexities, funders and evaluators should not emphasize “attribution” which forces people to look for more arbitrary measures of success. They should instead embrace a perspective which evaluates the leadership’s program contribution (kind, quantity, and causal pathway) which enables program implementers and evaluators to focus attention where they are really hoping to see a difference.

### Evaluation Design and Approach

Typically, stronger impact evaluation designs are considered to be those which compare the results of the program to a counterfactual, a comparison which is usually done through experimental or quasi-experimental research designs [17,18]. However, experimental and quasi-experimental designs may not address the emergent nature of complex programs (where program design and intended outcomes often change in response to contextual, organizational, and political factors). They also often fail to identify multiple, nonlinear, and recursive causal pathways and unintended outcomes [19]. Because of the multiple confounders and influencers at both the individual and societal levels, sustaining a meaningful comparison group over the long term may be challenging (over time the “counterfactual” may be exposed to very similar influences as the intervention of interest, creating a contamination effect). Therefore, revisiting causal mechanisms, identifying data points necessary to understanding the processes, and connecting the program’s key components will continue to be key to identifying whether and how longer/broader term impacts of the intervention are occurring.

## Vision for the Future

The field of global health is in agreement that leadership matters for strengthening health programs and improving public health outcomes. In this article we argue that evaluation plays an important role in understanding how leadership development takes place and how it contributes to strong institutions and better health. Making the case for investments in leadership development requires solid measurement and an on-going focus on evaluation that can credibly link investments to longer-term impacts. Ongoing attention to leadership evaluation is critical to the case for leadership development as an essential component of public health. Here we highlight gaps that need to be addressed and some ways forward.

For individuals who pass through leadership development programs, there may be a tension between what their organizations measure (in terms of performance outcomes, remuneration and incentives) and the developmental outcomes that leadership programs may espouse. These organizational performance outcome measures may fail to include non-quantifiable metrics and fundamentally be less concerned with how leadership emerges and how it is mutually constructed. The result is that organizations are likely to under-value the contributions of leadership training programs.

The first crucial step towards a strategy for success is for programs to clearly build, articulate, and share their theories of change. Stronger theories of change can support leadership development in ways that go beyond evaluative functions. They help develop and confirm a program’s leadership ideology so there is a consistent approach across delivery and evaluation. They also help identify the pathways and potential tensions that are believed to drive leadership development within a particular organizational and institutional context. They also help build the case to funders by telling a clear story of how leadership matters and what kinds of outcomes funders might expect from their investments.

Given that we are now at a critical juncture for global health, where the conditions and needs for effective healthcare delivery are changing, addressing the ‘interpretation of leadership’ and ‘drivers of leadership’ evaluation gaps is vital. Left unaddressed, these evaluation gaps can hinder the ability to develop leaders with the capacity to establish and deliver both population-based and individually targeted solutions, which will be required from leaders in public health. Bridging the ‘interpretation of leadership’ evaluation gap requires recognition that while there are many definitions of leadership, leadership programs need to clearly identify the type(s) of leadership roles and responsibilities in global public health they are supporting.

Making the case for investments in leadership development requires an approach to impact measurement that moves beyond individual leadership outcomes. Moving beyond individual outcomes requires a better understanding of how leadership development affects the performance of host organizations, as well as how feedback loops and network interactions can amplify the effects of original investments to improve community and population-level outcomes. Making this case requires funding for evaluations that are multi-method, longer-term and that focus on identifying causal effects where possible, yet pay close attention to credible contribution to organizational and societal outcomes.

## Conclusion

A thoughtful M&E strategy has the ability to not only harness meaningful indicators that seek to quantify the outcomes of a global health leadership training program, but also, when nested within a theory of change, allows for an understanding of the causal pathways that contribute to success. A holistic approach to M&E requires capture of both short and long-term indicators across socio-ecological levels, some of which are proposed in this paper. There is a greater need in the field of global health leadership training, however, to support the development and measurement of impact indicators that span beyond the program and the individual trainee, to also encompass the organization and community where that the trainee works. Leadership measurement that truly captures impact requires funding to support evaluation long after the training program is completed. Such an investment is necessary if donors are to identify and thus support programs that have the ability to make long-lasting sustainable impact.
